# Preparation and characterization of *Pistacia atlantica* oleo-gum-resin-loaded electrospun nanofibers and evaluating its wound healing activity in two rat models of skin scar and burn wound

**DOI:** 10.3389/fphar.2024.1474981

**Published:** 2024-11-25

**Authors:** Ghobad Mohammadi, Mosayyeb Safari, Masoud Karimi, Amin Iranpanah, Mohammad Hosein Farzaei, Sajad Fakhri, Javier Echeverría

**Affiliations:** ^1^ Pharmaceutical Sciences Research Center, Health Institute, Kermanshah University of Medical Sciences, Kermanshah, Iran; ^2^ Student Research Committee, Kermanshah University of Medical Sciences, Kermanshah, Iran; ^3^ Departamento de Ciencias del Ambiente, Facultad de Química y Biología, Universidad de Santiago de Chile, Santiago, Chile

**Keywords:** burn wound, wound healing, excision, skin scar, *Pistacia atlantica* gum, nanofibers

## Abstract

**Background:**

A growing body of research is dedicated to developing new therapeutic agents for wound healing with fewer adverse effects. One of the proceedings being taken today in wound healing research is to identify promising biological materials that not only heal wounds but also vanish scarring. The effectiveness of nanofibers like polyvinyl alcohol (PVA), in improving wound healing can be related to their unique properties. *Pistacia atlantica* Desf. subsp. *kurdica* (Zohary) Rech. f. (*PAK*) [Anacardiaceae], also known as “Baneh” in traditional Iranian medicine, is one of the most effective herbal remedies for the treatment of different diseases like skin injuries due to its numerous pharmacological and biological properties, including anti-inflammatory, antioxidant, and anti-bacterial effects.

**Purpose:**

Our study aimed to evaluate the wound-healing activity of nanofibers containing PVA/*PAK* oleo-gum-resin in two rat models of burn and excision wound repair.

**Material and Methods:**

PVA/*PKA* nanofibers were prepared using the electrospinning method. Scanning electron microscope (SEM) images and mechanical properties of nanofibers were explored. Diffusion and releasing experiments of nanofibers were performed by the UV visible method at different time intervals and up to 72 h. The animal models were induced by excision and burn in Wistar rat’s skin and the wound surface area was measured during the experiment for 10 and 21 days, respectively. On the last day, the wound tissue was removed for histological studies, and serum oxidative factors were measured to evaluate the antioxidant properties of the PVA/*PKA*. Data analysis was performed using ImageJ, Expert Design, and statistical analysis methods.

**Results and discussion:**

PVA/*PKA* nanofibers were electrospun at different voltages (15, 18, and 20 kV). The most suitable fibers were obtained when the nozzle was positioned 15 cm away from the collector, with a working voltage of 15 kV, and an injection rate of 0.5 mm per hour, using the 30:70 w/v *PKA* gum. In the SEM images, it was found that the surface tension of the polymer solution decreased by adding the gum and yield thinner and longer fibers at a voltage of 15 kV with an average diameter of 96 ± 24 nm. The mechanical properties of PVA/*PKA* nanofibers showed that the presence of gum increased the tensile strength and decreased the tensile strength of the fibers simultaneously. *In vivo* results showed that PVA/*PKA* nanofibers led to a significant reduction in wound size and tissue damage (regeneration of the epidermal layer, higher density of dermal collagen fibers, and lower presence of inflammatory cells) compared to the positive (phenytoin and silver sulfadiazine) and negative control (untreated) groups. Wound contraction was higher in rats treated with PVA/*PKA* nanofibers. Additionally, antioxidative serum levels of catalase and glutathione were higher in the PVA/*PKA* nanofiber groups even in comparison to positive control groups.

**Conclusion:**

*Pistacia atlantica* oleo-gum-resin-loaded electrospun nanofibers potentially improve excision and burn models of skin scars in rats through antioxidative and tissue regeneration mechanisms.

## 1 Introduction

The skin, as the heaviest and largest organ in our body, constitutes approximately 15% of the total body mass and encompasses a surface area of about 1.5–2 m^2^ in adults (Richardson, 2003; Lai-Cheong and McGrath, 2017). It acts as a pivotal interface between the internal and external environments and acts as a protective barrier to protect the body against various environmental damages and infections. Additionally, it plays a crucial role in homeostasis, thermoregulation, metabolic, neurosensory, and immunologic functions (Naseri-Nosar et al., 2018; Heidari et al., 2019). Wounds emerge as a significant factor that disrupts the skin’s defensive capacity and exposes the body to protein and water depletion as well as infection. Wounds can occur due to scratching, burn, surgery, exposure to chemicals, abrasion, drug reactions, cold, and pressure, or as a consequence of diseases like psoriasis, eczema, and carcinomas, which can notably affect patients’ quality of life (Enoch and Price, 2004; Lai-Cheong and McGrath, 2017; Shpichka et al., 2019). Burn injury occurs subsequent to the skin being harmed by elevated temperature, electricity, chemical substances, or radiation. Severe complications from extensive or deep burns may occur, such as sepsis caused by bacterial infection, contraction of scar tissue after improper wound healing, or shock due to hypovolemia (Shpichka et al., 2019). The skin has also developed efficient, rapid, multifaceted, and dynamic mechanisms to close breaches in its protective barrier, which is collectively referred to as the wound healing response. Overall, this process involves four interconnected yet distinct biological processes including hemostasis, inflammation, proliferation, and remodeling. These phases collectively form the cascade of wound healing, and any deficiency within these phases can impede the body’s ability to heal wounds (Wilkinson and Hardman, 2020; Almadani et al., 2021). This intricate process encompasses a sequence of occurrences, diverse types of tissue, cells, and mediators including platelets, various growth factors (e.g., platelet-derived growth factor (PDGF), fibroblast growth factor (FGF), vascular endothelial growth factor (VEGF), transforming growth factor-β (TGF-β), etc.), oxidative stress factors (e.g., reactive oxygen species (ROS), catalase (CAT), glutathione peroxidase (GPx), nitric oxide (NO), etc.), inflammatory cells and mediators (e.g., macrophages, interleukins (ILs), tumor necrosis factor-α (TNF-α) interferons (IFNs), etc.), angiogenesis, cell proliferation, granulation, extracellular matrix (ECM) formation (e.g., proteoglycans, vitronectin, fibronectin, thrombospondin, and collagen III), and ultimately the formation of a scar, all working in harmony (Kurahashi and Fujii, 2015; Tottoli et al., 2020; Wilkinson and Hardman, 2020; Shaygan et al., 2021). In addition, growing evidence emphasizes the significance of targeting oxidative stress and inflammatory mediators to facilitate the process of wound recovery (Kurahashi and Fujii, 2015; Wilkinson and Hardman, 2020; Lopez et al., 2022; Wang et al., 2023).

Various strategies have been utilized for wound healing, encompassing methodologies such as cell therapy, chemical and herbal medicines, wound dressings, as well as laser therapy. The overall goal of all these treatment approaches is to facilitate prompt healing with minimal complications and simultaneously ensure cost-effectiveness (Pereira and Bártolo, 2016; Kolimi et al., 2022). In the context of wound healing, the selection of an ideal dressing plays a critical role. Effective wound dressing should prevent bacterial infiltration, have antibacterial properties, sufficient oxygen permeability, absorb wound secretions, and exhibit biological and structural characteristics similar to the extracellular matrix to accelerate wound healing (Selig et al., 2012; Dhivya et al., 2015; Connelly and McColl, 2016; Omar et al., 2017; Vowden and Vowden, 2017; Li et al., 2019). In creating new dressings, adding features such as anti-inflammatory effects, preventing cell death, even helping to heal cells, repairing nerve cells, and reacting quickly and intelligently to wound infection can be helpful (Dhivya et al., 2015). Conventional dressings (e.g., gauze, silver ion dressing, alginate dressing, hydrogel, etc.) have been used to cover wounds, prevent contamination, and create a suitable environment for wound healing (Naseri-Nosar et al., 2017; Alves et al., 2024). Recently, researchers have been trying to provide biocompatible and biodegradable nanofiber dressings that have unprecedented properties such as high surface area and high porosity to boost wound healing (Jeong et al., 2012). Nanofibers can be obtained through various techniques (Chen et al., 2017). The electrospinning process is the most common method due to its simplicity, cost-effectiveness, and flexibility (Barnes et al., 2007). Electrospinning nanofibers have attracted substantial attention due to their unique properties, such as exceptional strength, high surface area to volume ratio, controllable pore size, and high porosity (Haider et al., 2018; Al-Abduljabbar and Farooq, 2022). These exceptional properties make nanofibers highly appealing and significant. Given that nanofiber scaffolds exhibit behavior comparable to the extracellular matrix, they emerge as promising candidates for functional wound dressing materials and tissue engineering (Owida et al., 2022; Flores-Rojas et al., 2023; Jiang et al., 2023). Several agents have long been used to produce electrospun nanofibers as wound dressings such as polyvinyl alcohol (PVA), poly-ethylene oxide (PEO), gelatin, chitosan (CS), collagen, polycaprolactone, fibrinogen, and poly-lactic acid (Powell et al., 2008; Guo et al., 2020; Stojanov and Berlec, 2020). PVA is a water-soluble, non-toxic, and biodegradable synthetic polymer with good mechanical properties. The presence of hydroxyl groups in the PVA structure gives it the ability to form a gel network that facilitates the electrospinning process (Fahami and Fathi, 2018). Natural polymers such as tree gums (e.g., gum arabic, gum karaya, and gum kondagogu), gellan gum, alginate, tragacanth gum, guar gum, and CS have been reported to be successfully electrospun and mixed with PEO or PVA are important resources, and comprehensive reports on electrospinning of these polymers have been presented in various studies. The principal challenges facing electrospinning of these tree gums are high molecular weight, reduced solubility, swelling properties, and proper selection of solvent electrospinning systems (Bonino et al., 2011; Elsabee et al., 2012; Vashisth et al., 2014; Padil et al., 2016).

Traditional and ethnomedicine from different countries are highly regarded as valuable resources for discovering new drugs. Additionally, the plant kingdom has shown great potential in providing alternative treatments with fewer side effects and lower costs for the management or treating different diseases like skin disorders. This is due to the extensive variety of phytochemicals found in plants, which possess anti-inflammatory, antioxidant, and immunomodulatory effects (Bahramsoltani et al., 2017; Heidari et al., 2019). Wild pistachio (*Pistacia*) is a genus of the Anacardiaceae family. *Pistacia atlantica* Desf. subspecies *kurdica* (Zohary) Rech. f. (*PAK*), also known as the “Baneh” in Iran, is one of the *Pistacia* species that grow naturally in some countries such as Iran, Turkey, and Iraq (Bahramnejad, 2014; Gharazi et al., 2021). In the inner tissues of the “Baneh” tree, sap is present and is commonly collected from the bark pores during the summer season. This sap is frequently found in the western regions of Iran, including Kurdistan and Kermanshah. This sap is called *Pistacia atlantica* oleo-gum-resin and is locally known as “Saqez gum” (Gharazi et al., 2021). Different parts of *Pistacia*, including essential oils, oleo-gum-resin, and leaves are chemically identified and used to treat various human diseases (Gourine et al., 2010; Peksel et al., 2010; Gharazi et al., 2021). In Traditional Persian Medicine (TPM), *PAK* is utilized in the treatment of different diseases like skin wounds, eczema, peptic ulcer, dyspepsia, asthma, and kidney diseases (Amin, 2005; Aghili, 2009; Bozorgi et al., 2013). Moreover, *PAK* represented significant anti-inflammatory, antioxidant, wound healing, antibacterial, and antifungal activities in recent reports (Tohidi et al., 2011; Haghdoost et al., 2013; Hatamnia et al., 2014; Farahpour et al., 2015; Minaiyan et al., 2015; Shialy et al., 2015).

Therefore, the purpose of this study was to assess the efficacy of the nanofibers containing PVA/*PAK* oleo-gum-resin in promoting wound healing in two rat models of burn and excision wound repair. Additionally, the potential antioxidative and tissue regenerative impacts of the prepared nanofibers were also examined.

## 2 Material and methods

### 2.1 Chemicals and reagents

Acetic acid, Polyvinyl alcohol (PVA), ammonium molybdate, hydrogen peroxide (H_2_O_2_), 5, 5′-dithiobis-(2-nitrobenzoic acid) (DTNB), and xylazine were purchased from Merck Company (Germany). Ketamine was procured from Alfasan (Woerden, Netherlands). Phenytoin cream via Behvazan Company (Irán) and silver sulfadiazine 1% cream was provided by Sobhan Darou Company (Irán). All used compounds and chemicals were of analytical grade.

### 2.2 Plant material

The gum of *P. atlantica* Desf. subsp. *kurdica* (Zohary) Rech. f. [Anacardiaceae] was collected in June 2020 from the Kermanshah province, Iran. The plant was approved by Department of Pharmacognosy, Faculty of Pharmacy, Tehran University of Medical Science, and a voucher specimen (No. PMP-818) was deposited at the Herbarium of Faculty of Pharmacy, Tehran University of Medical Sciences, Tehran, Iran. 0.4 g of gum was suspended in 9.6 mL of acetic acid, and then it was filtered using Whatman No. 1 filter paper to obtain the gum solution (*PKA* gum).

### 2.3 Preparation of polymeric solution

To prepare a PVA solution, 0.8 g of the polymer was added to 9.2 mL of distilled water and stirred for 12 h. *PKA* gum was then added to the PVA solution at 30:70, 40:60, and 50:50 w/v and stirred at room temperature for 24 h.

### 2.4 Preparation of nanofibers by electrospinning method

After preparing polymer solutions containing *PKA* gum with concentrations of 30:70, 40:60, and 30:70 w/v, the desired experiments were conducted to achieve the desired nanofibers using an electrospinning device. In order to obtain the optimal nanofibers, various parameters of the electrospinning process, including material concentration (mentioned above), the distance between the nozzle and the collector, and the voltage applied were thoroughly examined. The electrospinning solutions were loaded into a 1 mL syringe (inner diameter of the needle = 0.8 mm) and fixed in a horizontal position on the syringe pump. Electrospinning was performed in ambient conditions (25°C and 30% relative humidity) using the following parameters: distances of 10, 15, and 20 cm and voltages of 15, 18, and 20 kV were investigated. A light microscope (Olympus CX23 light microscope, Dino-Lite camera, and DinoCapture 2.0 software) was employed to identify the most suitable fiber. The best nanofibers were obtained when the nozzle was positioned 15 cm away from the collector, with a working voltage of 15 kV, and an injection rate of 0.5 mm per hour, using the 30:70 w/v *PKA* gum.

### 2.5 Scanning electron microscope analysis

The diameter distribution and morphology of nanofibers were assessed using a scanning electron microscope (SEM), FEI Model Quanta 450 FEG, Hillsboro, OR, United States, with an operating voltage of 25 kV. To prepare the samples, they were fixed to an aluminum stub and subsequently coated with a thin layer of gold under an argon atmosphere. The diameter range of nanofibers was analyzed with ImageJ (National Institutes of Health, United States). The average diameter of nanofibers was determined by measuring the diameter of 100 randomly selected fibers.

### 2.6 Release test

In order to determine the quantity of *PAK* released from the proper nanofiber, 100 mg of nanofiber was measured and placed within a cellulose membrane (Merck Millipore, Dialysis sacks, Avg. flat width 35 mm (1.4 in), MWCO 12,000 Da), containing 2 mL of phosphate buffer. This membrane was then submerged in a 50 mL solution of phosphate buffer at pH 7.2, which served as the release medium, and put in a shaker incubator (100 rpm at 25°C). This procedure was repeated three times and samples were collected at various intervals up to 72 h. The absorbance of the collected samples was subsequently measured using a UV-visible apparatus at a wavelength of 260 nm. Then, the average absorption of the samples was plotted at different times, and based on that, the *PAK* release curve was obtained.

### 2.7 Mechanical properties of nanofibers

The mechanical properties of the PVA and PVA/*PAK* gum were assessed using a mechanical tensile testing device (STM-1 DBBP-100, South Korea). The nanofibers were cut to dimensions of 40 mm × 10 mm and related mechanical properties were examined at room temperature. The tensioning speed was 1 mm/min.

### 2.8 Animals and ethical considerations

Adult male Wistar rats (weighing 200 ± 20 g) that were free from any skin disorders and infections were obtained from the pet of Kermanshah University of Medical Sciences, School of Pharmacy. The rats were maintained under specific conditions including a temperature of 22–25°C, a 12:12 h light/dark cycle, free access to rodent food, and tap water. The cages were cleaned once per week. Tissue samples were obtained by sacrificing the animals. All animal procedures were approved by the animal ethics committee of Kermanshah University of Medical Sciences, Iran (IIR.KUMS.REC.1398.1048), and were carried out in compliance with relevant guidelines and regulations for the care and use of laboratory animals at the Institute.

### 2.9 Creation of excision wound and burn injury

The rats were anesthetized via intraperitoneal (i.p.) injection of a combination of ketamine (80 mg/kg) and xylazine (10 mg/kg). Following this, the dorsal surface of the rats was shaved and sterilized. To create an excision wound, a section of skin was removed with a scalpel blade to create a 20 mm diameter excision wound on the back of each rat. A burn wound was created by applying an aluminum rod (20 mm diameter) on the shaved area of rats which was heated to 100°C for 10 s. Animals were housed individually (one in a cage) and treatment started 24 h after the introduction of wounds and continued to day 10, and 21 for excision and burn models, respectively.

### 2.10 *In vivo* assessment of wound healing activity of PVA/*PAK* gum nanofibers

Forty-eight male Wistar rats, weighing 200 ± 20 g, were randomly allocated into eight different groups of six (*n* = 6).

#### 2.10.1 Excision wound healing activity

The animals were randomly segregated into four groups of six: (1) Control (negative control group, normal saline, 1 mL for each rat), (2) Phenytoin cream 1% (positive control group, 1 g for each rat), (3) PVA nanofibers (polymer only group), and (4) PVA + Extract (PVA/*PAK* gum nanofibers). All groups were treated once daily for 10 days and nanofibers were applied topically in the volume to fully cover the 20 mm wounds on the back of each rat. The wound was dressed by a regular dressing and changed every 24 h. On the 11th day, the rats were sacrificed, and prior to their sacrifice, blood samples were collected from their aorta to evaluate the serum levels of the CAT and GSH, which are indicative of oxidative stress.

#### 2.10.2 Burn wound healing activity

The animals were randomly segregated into four groups of six: (1) Control (negative control group, normal saline, 1 mL for each rat), (2) silver sulfadiazine cream 1% (positive control group, 1 g for each rat), (3) PVA nanofibers (polymer only group), and (4) PVA + Extract (PVA/*PAK* gum nanofibers). Rats were treated once daily for 10 days and nanofibers were applied topically in the volume to fully cover the 20 mm wounds. The nanofibers served as a topical patch or dressing and were directly applied in the appropriate dimensions onto the wound area. The wound dressed by a regular dressing and changed every 24 h. On the 22nd day, the rats were sacrificed, and prior to their sacrifice, blood samples were collected to evaluate the serum levels of the CAT and GSH, which are indicative of oxidative stress.

### 2.11 Wound size assessment

To assess wound contraction, a digital camera was employed to capture photographs. The acquired images underwent analysis utilizing ImageJ software to determine the size of the wound area. The rate of wound closure was subsequently expressed as a percentage, reflecting the reduction in size compared to the initial measurement on day zero. This rate was computed using the following formula (Shaygan et al., 2021):

$$\text{Wound contraction }\left(\%\right)=\left(\text{wound size of the induction day}-\text{wound }\text{size }\text{of the specific day }\left(\text{days after treatment}\right)\right))/\text{wound size of the induction day}\times 100.$$

### 2.12 Histopathological analysis

For histopathological assessments, the rats underwent anesthesia by i.p. administration of thiopental sodium. Skin tissue samples from the wound areas were collected and fixed with a 10% formalin. After tissue preparation, 7 μm thick tissue sections were stained with hematoxylin and eosin (H&E). The samples were visualized using the Olympus CX23 light microscope, Dino-Lite camera, and DinoCapture 2.0 software. The histopathological investigations were carried out by an experimenter who was blinded to the study.

### 2.13 Catalase assay

CAT activity was assessed in order to evaluate the level of antioxidants, following the procedure outlined by Aebi (1984). In brief, 20 µL of serum samples were combined with 100 µL of H_2_O_2_ (65 mM) in 96-well plate wells. The mixture was then incubated at room temperature for 4 min. To stop the reaction, 100 µL of ammonium molybdate (32.4 mM) was added, resulting in the formation of a yellow molybdate and H_2_O_2_ complex. The absorbance of the sample was subsequently measured at a wavelength of 405 nm using an ELISA reader (Iranpanah et al., 2024). Each assay was conducted in triplicate based on the following formula:

$$\text{Concentration difference }\left(\%\right)=\left\{\left(C_{\text{Health}}‐C_{\text{Sample}}\right)/C_{\text{Health}}\right\}\times 100$$

### 2.14 Glutathione assay

The antioxidant level was assessed by measuring the reduced GSH levels using the Ellman method established in 1959. This method involves the oxidation of GSH through DTNB, resulting in the formation of the yellow derivative 5′-thio-2-nitrobenzoic acid (TNB), which can be measured at a wavelength of 412 nm. To perform the assay, 40 μL of serum samples were mixed with 50 μL of phosphate-buffered saline (PBS, 0.1 M) with a pH of 7.2 in each well. Subsequently, 100 μL of the Ellman or reagent 5′-dithiobis-(2-nitrobenzoic acid) (DTNB) was added. The plate was then incubated at a temperature of 37°C for 10 min. Following the incubation period, the optical density (OD) of the mixture was measured at a wavelength of 412 nm using an ELISA reader (Iranpanah et al., 2024). Each assay was conducted in triplicate to ensure the accuracy and consistency of the results (Rahman et al., 2006). Each assay was conducted in triplicate based on the following formula:

$$\text{Concentration difference }\left(\%\right)=\left\{\left(C_{\text{Health}}-C_{\text{Sample}}\right)/C_{\text{Health}}\right\}\times 100$$

### 2.15 Statistical analysis

All data were presented as mean ± standard error of the mean (SEM) and mean ± standard deviation (SD) for formulation evaluations. Repeated measures of one-way and two-way analysis of variance (ANOVA) were conducted, followed by Tukey’s *post hoc* analysis. In all analyses, a difference with *p* < 0.05 was regarded as significance.

## 3 Results

### 3.1 Morphological studies

**FIGURE 1:**
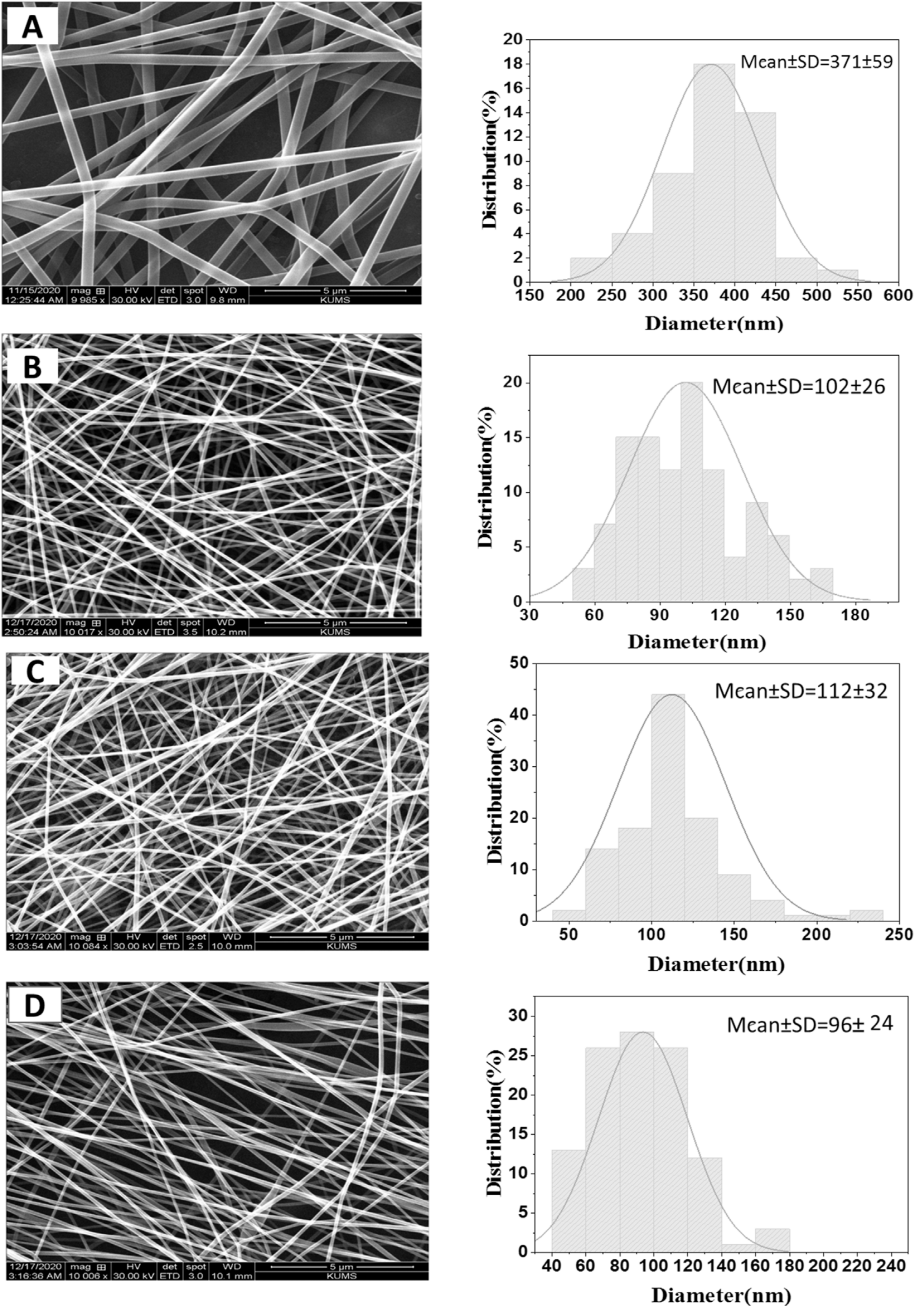
SEM images of PVA/*PAK* gum nanofibers at different voltages. **(A)** PVA, **(B)** PVA/*PAK* gum at 20 kV, **(C)** PVA/*PAK* gum at 18 kV, and **(D)** PVA/*PAK* gum at 15 kV.

PVA/*PAK* gum nanofibers were electrospun at 15, 18, and 20 kV voltages (Figure 1). The nanofibers were produced uniformly and without nuts at all voltage levels. Figure 1A showed the SEM image of PVA nanofibers without gum. The addition of PVA to the gum resulted in a reduction in viscosity and an enhancement in the electrospinning ability of the polymer-gum mixture. Furthermore, the incorporation of gum into PVA led to a significant decrease in the diameter of the polymer, indicating a reduction in the surface tension of the polymer solutions when gum was added. This reduction in surface tension allowed to produce finer and longer fibers at a constant voltage. Figures 1B–D showed PVA/*PAK* gum nanofibers at different voltages, with an average diameter ranging from 96 to 112 nm (Table 1). Among the various voltages, the PVA/*PAK* gum nanofibers fabricated at 15 kV were considered optimal due to their higher uniformity and fineness.

**TABLE 1:** The average diameter of PVA/*PAK* gum nanofibers at different voltages with a 5 µm magnification.

Nanofibers	Average diameter (nm)
PVA	371 ± 59
PVA/*PAK* gum at 20 kV	102 ± 26
PVA/*PAK* gum at 18 kV	112 ± 32
PVA/*PAK* gum at 15 kV	96 ± 24

### 3.2 Release test

**FIGURE 2:**
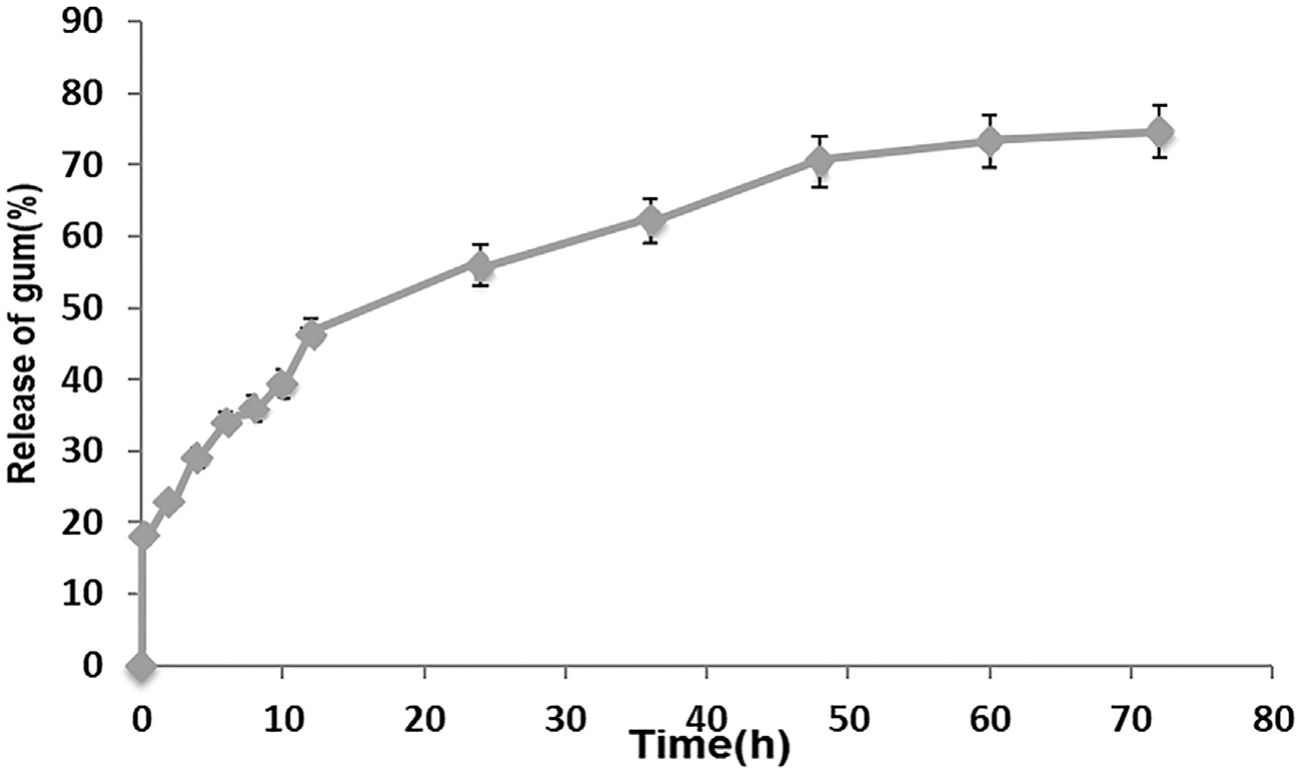
Release test. Release percentage of *PAK* from optimal PVA/*PAK* gum nanofibers (*n* = 3).

The release profile of *PAK* from the optimal PVA/*PAK* gum nanofibers at pH 7.2 in different times is represented in Figure 2. The findings indicate that the total release of *PAK* from nanofibers was 55.95% after 24 h and 74.60% after 72 h.

### 3.3 Mechanical properties of nanofibers

**FIGURE 3:**
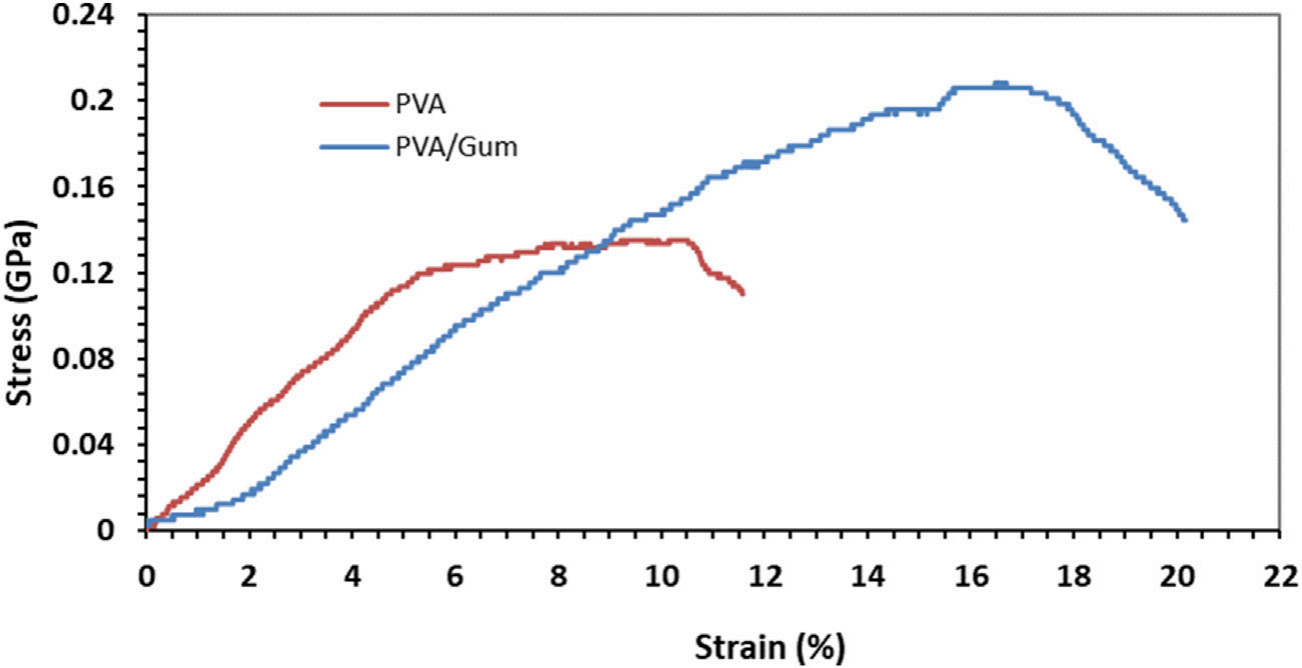
Mechanical test. Tensile stress–strain curves of PVA nanofiber and PVA/*PAK* gum nanofiber.

The stress-strain curve of electrospun PVA and PVA/*PAK* nanofibers obtained from the tensile test is shown in Figure 3. Based on the findings, both the modulus of elasticity and the tensile strength are higher in nanofibers with *PAK* gum compared to those without *PAK* gum. The percentage of elongation of PVA sample is 11.56% and its tensile strength is 0.13 GPa. In the PVA/*PAK* gum sample, the elongation percentage and tensile strength increased to 20.17% and 0.206 GPa, respectively. It seems that the presence of *PAK* gum has increased the elasticity and tensile strength of the nanofibers. This behavior can also be attributed to changes in the nanofiber diameter in the presence of *PAK* gum. As shown in the SEM images (Figures 1B–D), the presence of gum reduced the viscosity of the solution and, consequently, reduced the diameter of the samples. A smaller fiber diameter allows for better placement of the layered and fibrous structures along the fiber axis. This has a positive effect on the tensile strength of the sample (Doustgani, 2016).

### 3.4 Wound contraction rate in the excision wound injury

**FIGURE 4:**
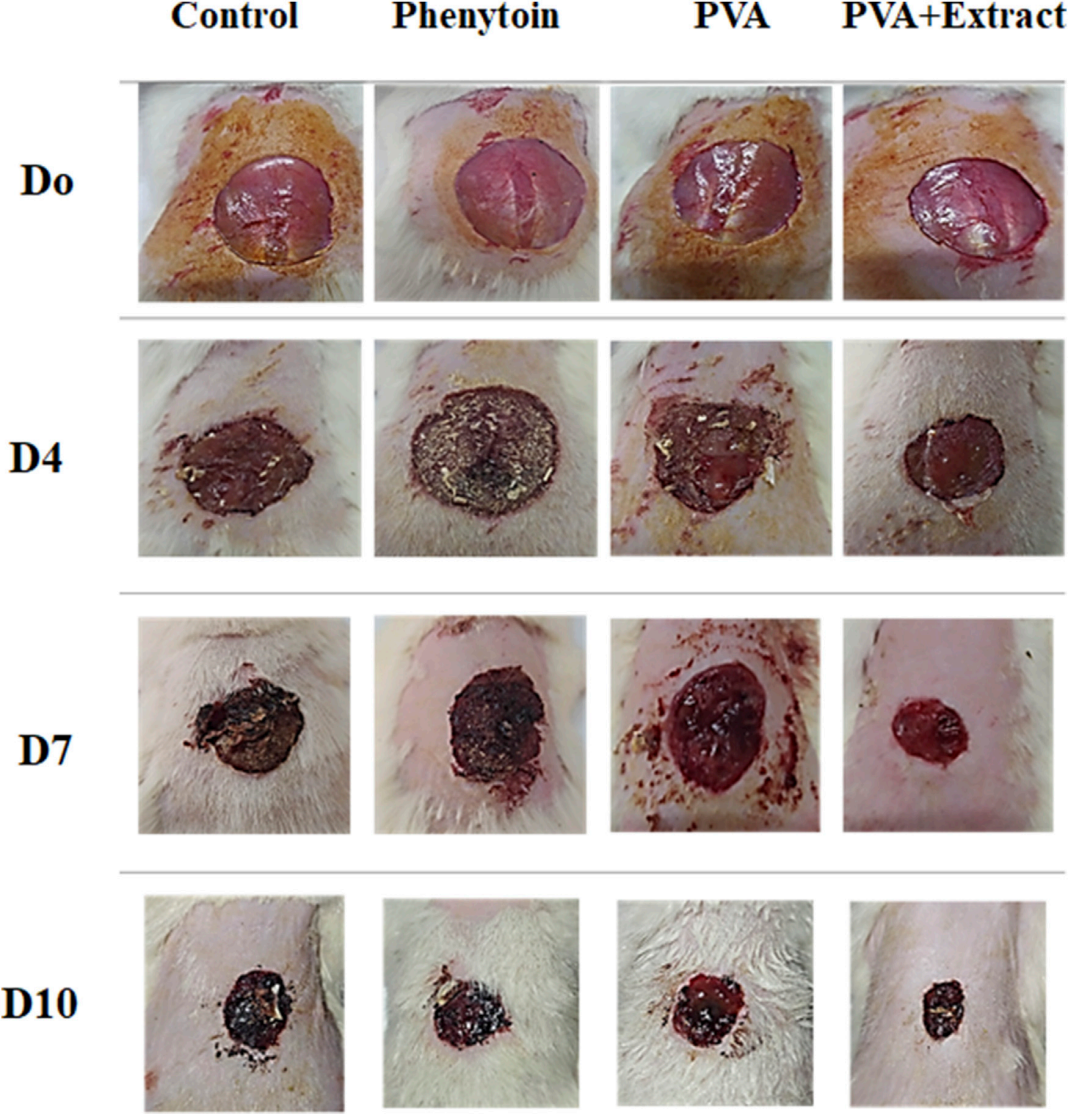
The macroscopic evaluation of wound size in the excision wound groups of receiving PVA/*PKA*. PVA, Polyvinyl alcohol; *PAK, Pistacia atlantica* Desf. subspecies *kurdica* (Zohary) Rech. f.

**FIGURE 5:**
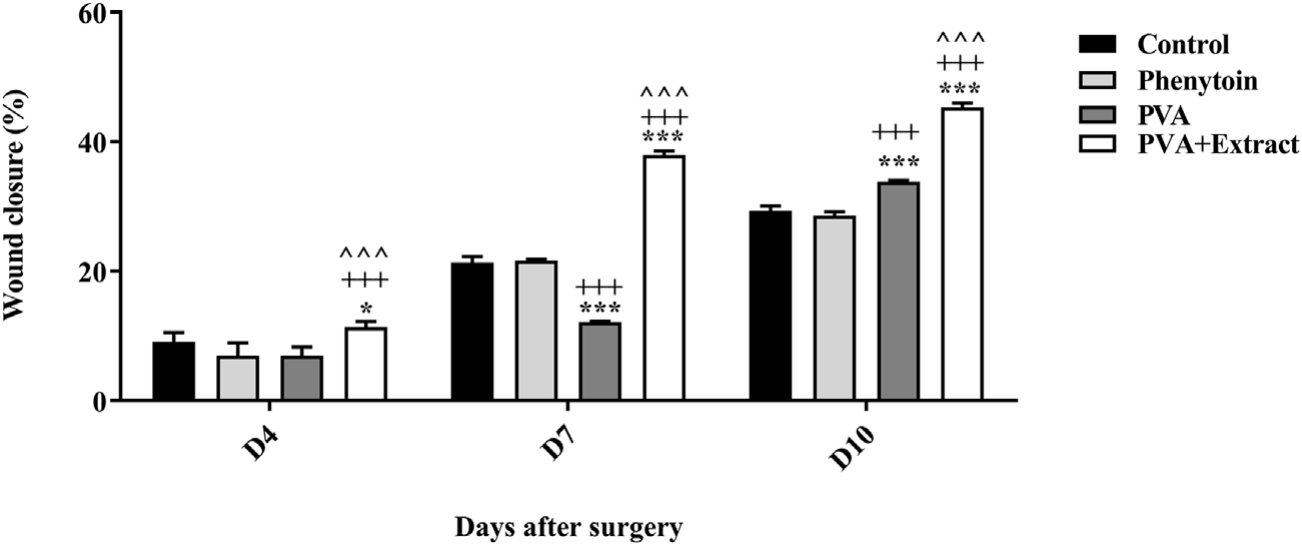
Wound closure rate of PVA/*PKA* and various treated groups on different days following the surgical procedure in the excision wound groups. *: *p* < 0.05, and ***: *p* < 0.001, vs. control group (normal saline); +++: *p* < 0.001, vs. phenytoin group; ^^^: *p* < 0.001 indicated a significant difference between PVA and PVA + Extract.

To evaluate the effectiveness of nanofibers in promoting the healing process of wounds, the size of the wounds was measured in various treated groups on days 0, 4, 7, and 10. According to the findings, rats whose wounds were treated with PVA/*PAK* gum nanofibers exhibited a notable decrease in wound size in comparison to the groups that received normal saline, phenytoin, and polymer after day 10. Consequently, the wound healing capability of PVA/*PAK* gum nanofibers was remarkably higher than other treated groups across all the assessed time intervals. Figure 4 illustrated the macroscopic trends of wound healing, and Figure 5 presented associated statistical results.

### 3.5 Wound contraction rate in the burn wound injury

**FIGURE 6:**
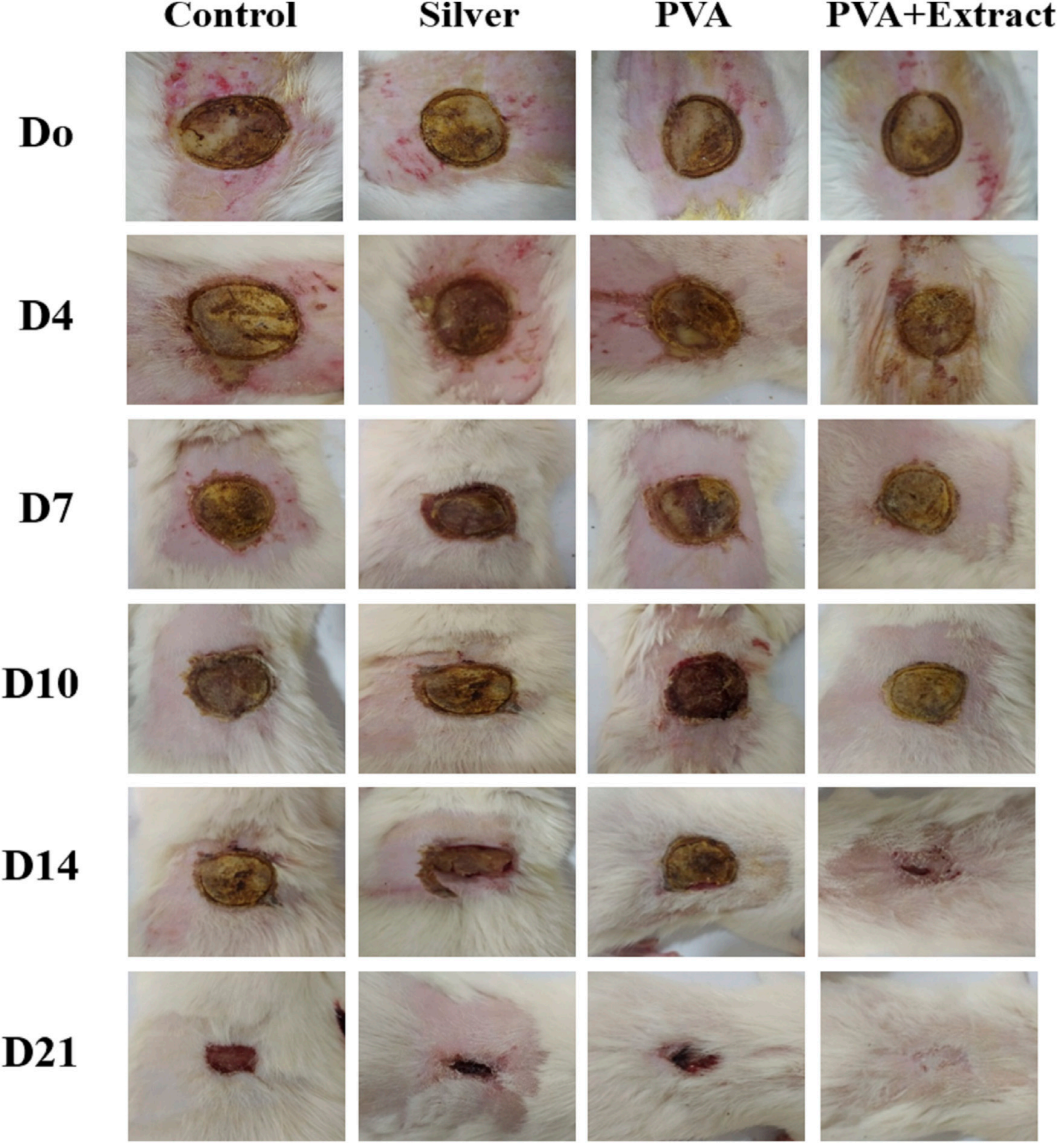
The macroscopic evaluation of wound size in the burn wound groups receiving PVA/*PKA*. PVA: Polyvinyl alcohol; *PAK*: *Pistacia atlantica* Desf. subsp. *kurdica* (Zohary) Rech. f.

**FIGURE 7:**
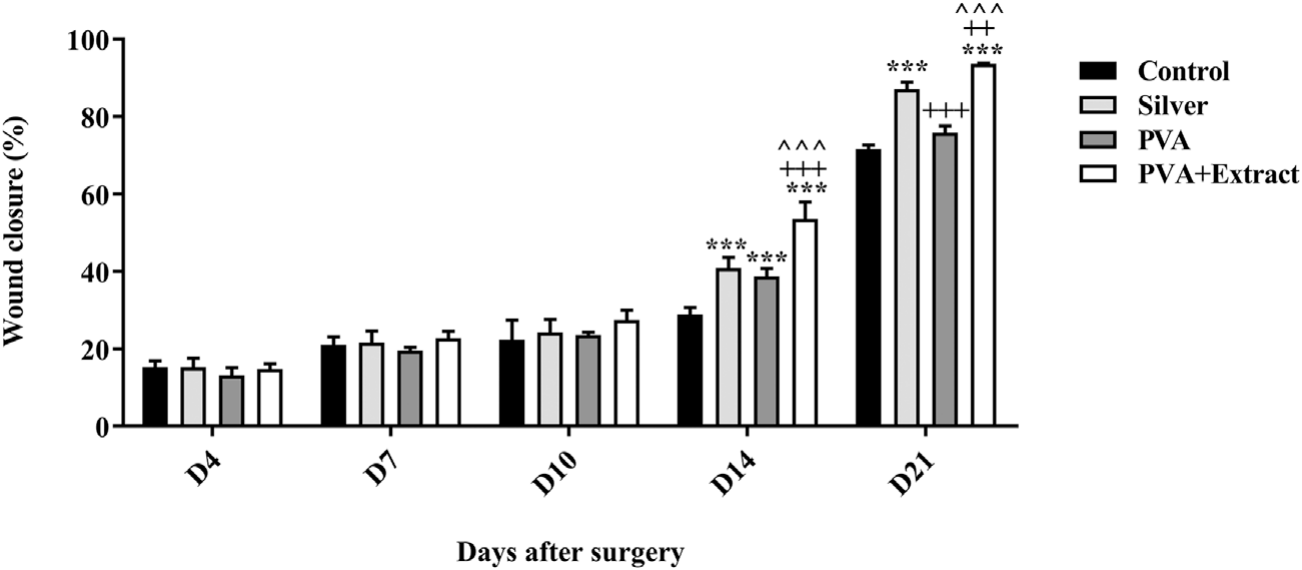
Wound closure rate of PVA/*PKA* and various treated groups on different days following the surgical procedure in the burn wound groups. ***: *p* < 0.001, vs. negative control group (normal saline); ++: *p* < 0.01, and +++: *p* < 0.001, vs. silver sulfadiazine; ^^^: *p* < 0.001 represented a considerable difference between PVA and PVA + Extract.

The objective of this study was to assess the efficacy of nanofibers in facilitating the wound healing process. To accomplish this, the size of the wounds was measured in different treated groups on days 0, 4, 7, 10, 14 and 21. Based on the outcomes, rats whose wounds were subjected to PVA/*PAK* gum nanofibers displayed a conspicuous reduction in wound size as compared to the groups that received normal saline, silver sulfadiazine, and polymer after day 21. Consequently, the wound healing potential of PVA/*PAK* gum nanofibers was significantly greater than that of other treated groups throughout all the evaluated time intervals. Figure 6 demonstrated the macroscopic trends of wound healing, and Figure 7 presented associated statistical results.

### 3.6 Histopathological analysis

**FIGURE 8:**
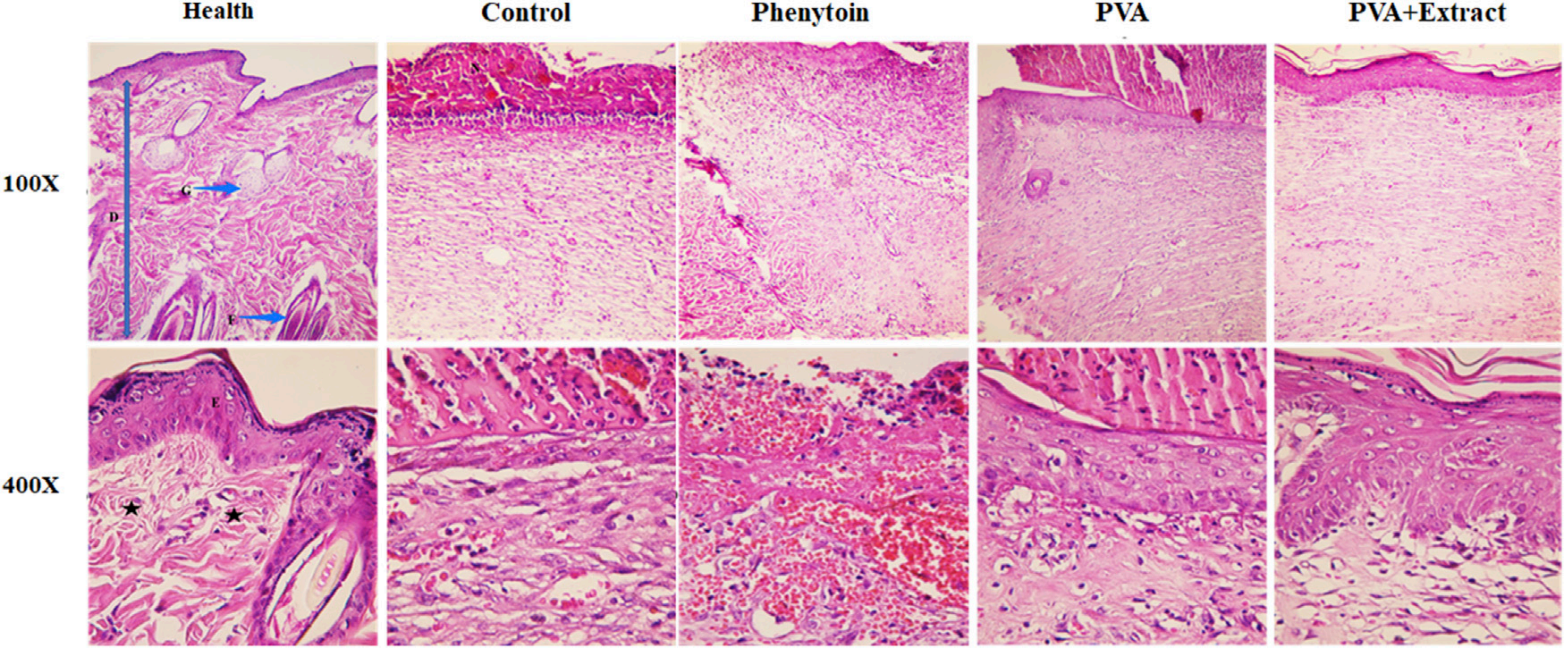
Histopathological evaluation of skin in the excision wound groups following receiving PVA/*PKA*. Skin sections in the different groups (H&E). (Health) ×100, and ×400: normal healthy skin is a thick tissue anatomically encompassing two main sections, namely, the epidermis and dermis. The epidermis is composed of a stratified squamous epithelium that is keratinized, whereas the dermis is constituted of a diverse array of appendages such as hair follicles, adipose glands, and multiple cellular components along with organized connective tissue patterns (asterisk). (Control) ×100, and ×400: magnifications of the negative control group skin tissue sections (normal saline). (PVA) ×100, and ×400: magnifications of the PVA-treated group skin tissue. (Phenytoin) ×100, and ×400: magnifications of the phenytoin-treated group. (PVA + Extract) ×100, and ×400: magnifications of the PVA/*PAK* gum-treated group skin tissue.

**FIGURE 9:**
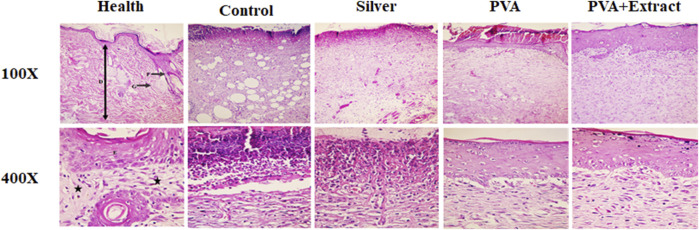
Histopathological evaluation of skin in the burn wound groups following receiving PVA/*PKA*. Skin sections in the different groups (H&E). (Health) ×100, and ×400: normal healthy skin is a thick tissue anatomically encompassing two main sections, namely, the epidermis and dermis. The epidermis is composed of a stratified squamous epithelium that is keratinized, whereas the dermis is constituted of a diverse array of appendages such as hair follicles, adipose glands, and multiple cellular components along with organized connective tissue patterns (asterisk). (Control) ×100, and ×400: magnifications of the negative control group skin tissue sections (normal saline). (PVA) ×100, and ×400: magnifications of the PVA-treated group skin tissue. (Silver) ×100, and ×400: magnifications of the silver sulfadiazine-treated group. (PVA + Extract) ×100, and ×400: magnifications of the PVA/*PAK* gum-treated group skin tissue.

Histological evaluation of tissue samples obtained from the wound site on the final day was performed by employing H&E staining. Figures 8, 9 presented magnifications of tissue sections derived from studied groups.

#### 3.6.1 Excision wound

Histological analysis indicated that healthy skin consists of three layers known as the epidermis, dermis, and hypodermis (Hofmann et al., 2023). Normal skin tissue sections (Figures 8, 9) are provided for comparison. The composition of healthy skin was characterized by its substantial thickness and includes various components such as keratinized stratified squamous epithelium, hair follicles, different types of connective cells, fat and sweat glands, multiple cellular elements, and clusters of connective fibers are seen. Conversely, the normal saline (negative control) group, exhibited disrupted tissue structure with a lack of epidermis formation on a large surface, and the dermis displayed an irregular and detached structure with a lack of appendages. Additionally, polymorphonuclears (PMNs) were observed in the wound area. The collagen fibers in the extracellular matrix were low-density and irregular, and no skin appendages were present (Figure 8). The group treated with PVA demonstrated improved skin tissue structure and in some cases, necrotic tissue and inflammatory cell accumulations were observed (Figure 8). However, the groups treated with PVA/*PAK* gum nanofibers and phenytoin (positive control) (Figure 8), represented significantly reduced skin tissue damage compared to the normal saline group. The wrinkled epidermis displayed remarkable regeneration and the dermis showed a high density of collagen fibers within the connective tissue. Additionally, normal cells were present, and there was a noticeable decrease in the infiltration of inflammatory cells. In the group treated with PVA/*PAK* gum nanofibers, the epidermal tissue was formed and folded, and the dermis had the same structure as healthy skin.

#### 3.6.2 Burn wound

In the group that received normal saline as a negative control, there was a disruption in tissue structure, a lack of formation of the epidermis on a large surface area, and an irregular and detached structure of the dermis with a lack of appendages. Furthermore, PMNs were visible in the wound area. Additionally, the extracellular matrix exhibited low-density and irregular collagen fibers and no skin appendages were observed (Figure 9). In the PVA treated group, skin tissue structure improved and in some cases, necrotic tissue and accumulations of inflammatory cells were noted (Figure 9). However, in the groups treated with PVA/*PAK* gum nanofibers and silver sulfadiazine (positive control) (Figure 9), the extent of skin tissue damage was significantly reduced in comparison to the normal saline group. The wrinkled epidermis exhibited significant regeneration. Moreover, the dermis displayed a high density of collagen fibers from connective tissue, along with the presence of normal cells and a notable decrease in the infiltration of inflammatory cells. Furthermore, in the PVA/*PAK* gum nanofibers treated group, the epidermal tissue was formed and folded, and the dermis had the same structure as healthy skin.

### 3.7 Catalase assay

**FIGURE 10:**
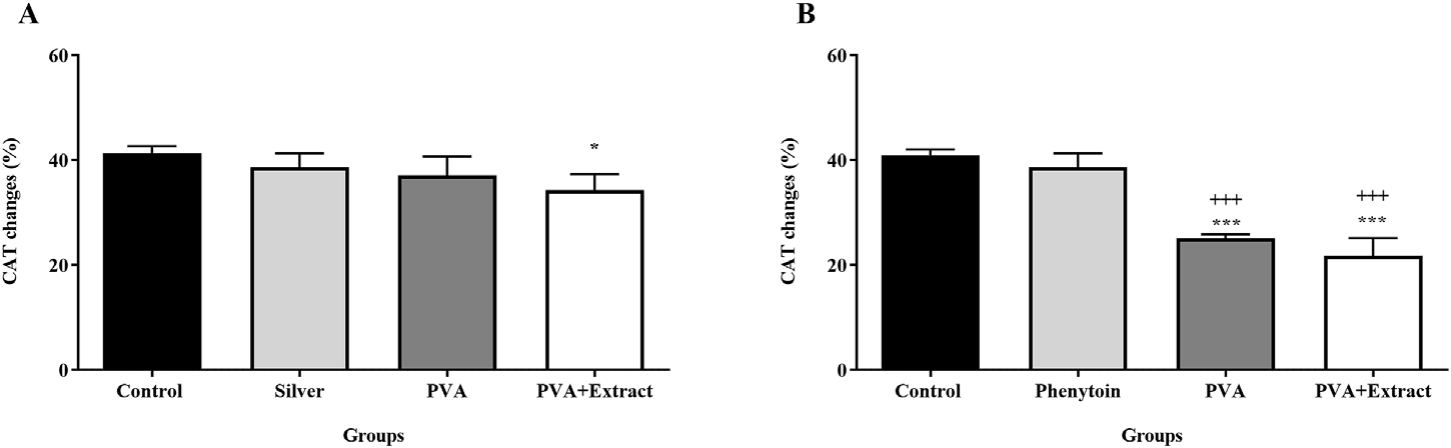
Catalase assay. Measurement of catalase by hydrogen peroxide assay in the burn wound **(A)** and excision wound **(B)** groups. Treatment with PVA/*PAK* gum exhibited a significant increase of serum CAT levels when compared to the control group. The data are reported as mean ± SEM (*n* = 3). Repeated measures one-way ANOVA followed by Tukey *post hoc* tests were used for data analysis. *p < 0.05, and ***p < 0.001 vs. negative control group; +++: *p* < 0.001, vs. silver sulfadiazine **(A)** and phenytoin **(B)** groups.

The concentration of catalase present in the serum is considered to possess antioxidant characteristics, and an increased concentration of catalase is deemed favorable. It was found that PVA and PVA/*PKA* notably increased CAT activity compared to the negative control group (untreated) in the burn group (*p* < 0.05). Additionally, in the excision model PVA/*PKA* significantly increased CAT in comparison to phenytoin, as positive control group (Figure 10).

### 3.8 Glutathione assay

**FIGURE 11:**
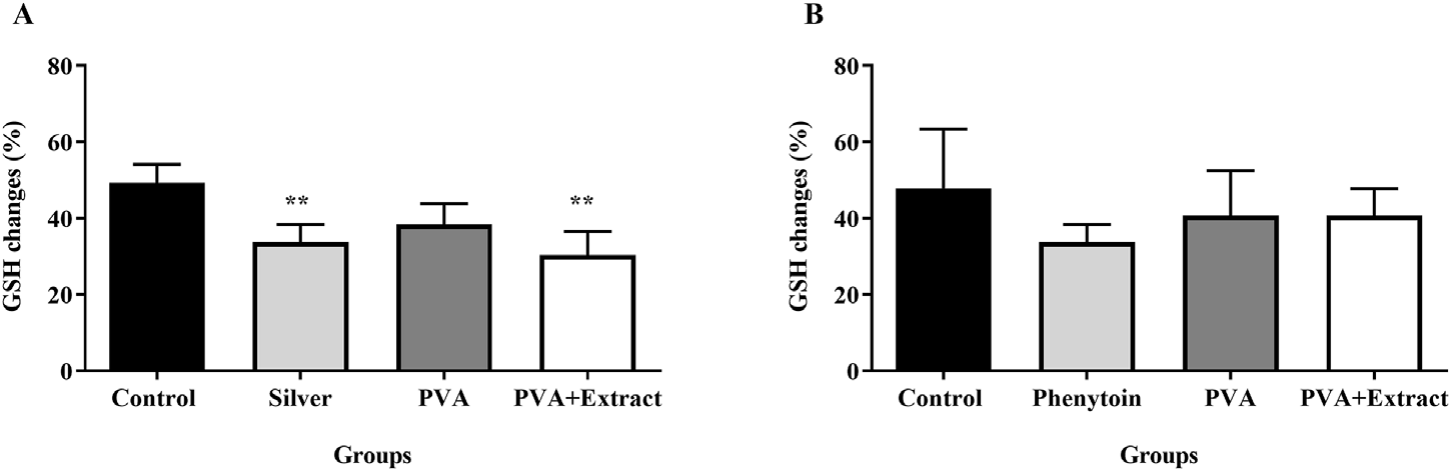
Glutathione assay. Effects of PVA/*PAK* gum on serum GSH levels in the burn wound **(A)** and excision wound **(B)** groups. Treatment with PVA/*PAK* gum resulted in a notable increase in GSH levels when compared to the normal saline group. The data are reported as mean ± SEM (*n* = 3). Repeated measures one-way ANOVA followed by Tukey *post hoc* tests were used for data analysis. **p < 0.01 vs. negative control group.

Glutathione, an essential enzyme with antioxidant properties, was analyzed through a glutathione assay and the results are displayed in Figure 11. The comparative assessment of antioxidant levels between the groups demonstrated the effectiveness of PVA/*PKA* in regulating the levels of GSH in the excision group in compare to control (untreated) group (*p* < 0.01).

## 4 Discussion

In the current study, PVA/*PKA* nanofibers were synthesized by the electrospinning method, and *PAK* gum was loaded on the surface of nanofibers in different concentrations (30:70, 40:60, and 50:50 w/v). According to the results, the best nanofiber with *PAK* gum was obtained (30:70 w/v) and were used for wound healing in rats. Other concentrations led to the production of irregular and unsuitable nanofibers. Based on the SEM images, *PAK* gum has been successfully loaded on optimal nanofiber. High tensile strength, ease of use, and handling of materials used for wound dressing are very important features of these nanofibers. For this reason, the tensile strength of the prepared nanofiber was investigated, and based on the results, the tensile strength of this nanofiber is high and acceptable. We exhibited the wound-healing efficacy of the *PAK* gum nanofibers in a rat model of excision and burn wound injury. According to wound closure results, *PAK* gum nanofibers have the potential to speed up the wound healing process in comparison to the negative (untreated) and positive (phenytoin and silver) controls, in both excision and burn wound injuries. Histopathological evaluation confirmed wound healing activity of *PAK* gum nanofibers, as well through regeneration of the epidermal layer, higher density of dermal collagen fibers, and lower presence of inflammatory cells. Additionally, we showed the antioxidant capabilities of the *PAK* gum nanofibers through CAT and GSH assay. Our study displayed that *PAK* gum nanofibers could enhance levels of serum GSH and CAT, indicating its systemic antioxidant activity that contributes to the promotion of wound repair.

From the mechanistic point of view, the wound-healing activity of *PAK* gum nanofibers could be attributed to the presence of diverse constituents like phenolic compounds and terpenoids that are able to speed up wound healing and exhibited antioxidant properties. According to our results, Haghdoost et al. (2013) demonstrated the wound healing potential of the *P. atlantica* resin extract by improving the angiogenesis and enhancing FGF and PDGF concentration, in the rat model of skin burn injury. They also presented *α*-pinene as one of the active compounds of essential oil of resin. By targeting VEGF, the resin oil of *P. atlantica* showed antioxidant effects in treating skin burn (Shahouzehi et al., 2018). In another study by Hamidi and colleagues, the cutaneous wound-healing activity of the topical *P. atlantica* gel was investigated. The gel with a concentration of 10%, decreased the total lymphocyte count and improved total fibrocyte count, number of blood vessels, and re-epithelialization. These effects were observed in terms of wound closure, histological analysis, and oxidative stress biomarkers (Hamidi et al., 2015; 2017). To evaluate the anti-inflammatory effect of *P. atlantica* volatile oil*,* alpha-pinene was found as the main constituent and reduced colitis and myeloperoxidase activity in rats (Minaiyan et al., 2015). Furthermore, some studies represented notable wound healing, antimicrobial and antifungal effects of *P. atlantica* extracts and found to be effective in scavenging and decreasing the superoxide anions *in vitro* (Benhammou et al., 2008; Tohidi et al., 2011). In another *in vitro* study *P. atlantica* was also presented as a natural anti-fungal agent against different fungal species (Shialy et al., 2015)**.** Employing high-performance liquid chromatography method, Hatamnia et al. showed that the highest antioxidant activity of *PAK* was attributed to related phenolic content, including vanillic acid, sinapic acid, and *p*-hydroxybenzoic acid (Hatamnia et al., 2014). In another study, topical administration of *P. atlantica* increased upregulated mast cells infiltration and hydroxylproline content, accelerated proliferation phase, while lowered RNA damage (Farahpour et al., 2015). Similarly, the antioxidant activity of *PAK* provided by Rahman in 2018 (Rahman, 2018). Therefore, these effects could be another reason for the wound healing effects of *P. atlantica*. Considering the antioxidant, anti-inflammatory and antimicrobial effects of *PAK*, as well as the advantages of using nanofiber wound dressings, the PVA/*PAK* gum nanofibers can serve as a natural treatment for the treatment of different types of wounds. In line with our results, Rajati et al. (2023) showed that a nanofiber-hydrogel composite from green synthesized Ag nanoparticles embedded to PVA hydrogel and *P. atlantica* gum nanofiber had the therapeutic potential in wound dressing.

Generally, PVA has shown potential and is widely used for wound healing because of its small-molecule permeability, soft consistency, transparency, low interfacial tension, and antibiotic release control (Saraiva et al., 2023). However, tacky mechanical properties restrict their potential as wound dressings (Song et al., 2021). Studies have shown that PVA can be cross-linked with Ca^2+^ or other agents, thereby improving biological and physicochemical properties in wound healing. In similar studies, the anti-bacterial effects of formulations containing PVA provided for wound healing with a high biocompatibility (Song et al., 2021).

In terms of pathology, wounds present a formidable clinical challenge due to the primary and secondary lesions that give rise to disease and high mortality rates (Natarajan et al., 2000; Panuncialman and Falanga, 2010). The healing process of wounds is characterized by its complexity and dynamic nature. It comprises four stages, namely, inflammation, proliferation (including re-epithelialization, granulation tissue formation, and neovascularization), hemostasis, and remodeling, that ultimately lead to the repair of dermis and epidermis tissues (Rousselle et al., 2019; Knoedler et al., 2023). Furthermore, recent reports have shed light on the importance of modulating inflammatory and oxidative stress mediators to facilitate the process of wound healing. Oxidative stress refers to an internal imbalance between the body’s prooxidants and antioxidants. During oxidative stress, the production of ROS increases, and these play a crucial role in wound healing. Low levels of ROS are involved in the regulation of numerous signal transduction pathways within cells (Dunnill et al., 2017; Qiu et al., 2021). Moreover, ROS modulates the wound-healing process through affecting inflammation, angiogenesis, extracellular matrix formation, and cell proliferation (Wang et al., 2023). To counter excessive oxidation reactions, the body produces antioxidant enzymes like superoxide dismutase (SOD), CAT, and GPx that neutralize ROS. For complex wounds that prove difficult to heal, the supplementation of antioxidants can aid in the protection of cells against oxidative damage and can enhance the process of wound healing (García-Sánchez et al., 2020; Qiu et al., 2021). The inflammatory response, which occurs shortly after an injury, primarily starts with the infiltration of PMN leukocytes or granulocytes and gives rise to the generation of free radicals at the site of inflammation. These free radicals can cause cellular damage and delay the healing process (Heidari et al., 2019).

Achieving faster wound healing with the least side effects is one of the main therapeutic aims of recent investigations. Wounds, as one of the main issues in the world, have long captivated the attention of medical researchers (Nuutila et al., 2014; Shaygan et al., 2021). Extensive investigation has been conducted, employing various treatment methods including chemical and medicinal therapies, herbal remedies, as well as physical approaches like laser therapy. The ultimate aims of recent studies are to discover effective, cost-efficient, less toxic, and rapid treatments for wound healing (Reinke and Sorg, 2012; Shaygan et al., 2021; Kolimi et al., 2022). The utilization of nanotechnology systems for delivering natural compounds holds great potential for enhancing the effectiveness of wound treatments. Various research groups worldwide are presently engaged in designing and manufacturing novel wound dressings (Ajith et al., 2023; Amutha Gokul et al., 2024; Nandhini et al., 2024). Nanofiber structures possess the ability to interact effectively with skin cells and their environment, expediting the wound-healing process (Liu et al., 2021; Ajith et al., 2023). Wound dressings play a pivotal role in managing wound healing by shielding the wound from external hazards and enhancing the healing process. The current market offers a range of common dressings including hydrogel, film, foam, sponge, and nanofiber membranes (Gupta et al., 2019; Hawthorne et al., 2021; Liu et al., 2021). Among these, electrospun nanofiber membranes illustrate a novel category of materials, characterized by their high surface-to-volume ratio, density, remarkable microporosity, and notable versatility (Fadil et al., 2021; Liu et al., 2021; Al-Abduljabbar and Farooq, 2022). These unique properties enable their utilization in various biomedical applications such as wound dressings, tissue engineering scaffolds, and drug delivery. Electrospun nanofiber wound dressings offer some advantages, including a structure and biological function that closely resemble the natural ECM, creating an optimal microenvironment for cell proliferation, migration, and differentiation (Liu et al., 2021; Ajith et al., 2023; Nandhini et al., 2024). Moreover, the extensive surface area and specific structure of the nanofiber membrane facilitate the effective loading of phytochemicals, antibacterial drugs, vitamins, growth hormones, and other biologically active ingredients (Ajith et al., 2023; Amutha Gokul et al., 2024). Therefore, the development and production of advanced and smart wound dressings capable of preventing wound infection and providing full support for wound healing can significantly reduce treatment costs and enhance patient wellbeing. Despite the availability of different topical preparations in the market, there is a noticeable lack of suitable medication for this purpose. Additionally, most of the currently available topical preparations or medications have negative adverse effects, toxicity, and primarily possess antimicrobial properties rather than wound repair effects (Heidari et al., 2019; Oliveira and Almeida, 2023). Modern medicine allocates nearly 1%–3% of its resources to wound repair, while traditional medicine proposed about 30% of its preparations to skin disorders, highlighting the high potential of traditional medicine in suggesting new drugs for wound care (Heidari et al., 2019). Natural products have the ability to induce wound-healing effects due to their diverse range of phytochemicals, such as phenolic compounds, terpenoids, alkaloids, fatty acids, and saponins (Vitale et al., 2022; Cedillo-Cortezano et al., 2024). These phytochemicals exert their effects at various stages of the healing process through a range of mechanisms, including the regulation of PMN, macrophages, ILs, IFNs, and TNF-α to induce an anti-inflammatory response and modulate ROS, CAT, SOD, and GSH levels to act as antioxidants. Additionally, they promote cell proliferation, enhance angiogenesis by upregulating TGF-β and VEGF levels, exhibit antimicrobial properties, and stimulate collagen synthesis (Vitale et al., 2022; Cedillo-Cortezano et al., 2024).

The most famous species of *Pistacia* [Anacardiaceae], namely, *P. atlantica* Desf., *Pistacia vera* L., *Pistacia khinjuk* Stocks, *Pistacia lentiscus* L., and *Pistacia terebinthus* L., are widely distributed in the Mediterranean and Middle Eastern regions (Mahjoub et al., 2018; Pekacar and Deliorman Orhan, 2022). The species *P. atlantica*, also known as wild pistachio, holds the most economical species and can be found in Iran, and has thee subspecies, k*urdica*, *mutica*, and *cabulica*. In Persian, *P. atlantica* is known as “Baneh,” Mt. Atlas mastic tree in English, Butm in Arabic, and Melengic in Turkish. In Iran, the oleo-gum-resin of *P. atlantica* is known as “Saqez gum” (Mahjoub et al., 2018). Extensive phytochemical studies on oleo-gum-resin of *P. atlantica* have revealed the presence of volatile compounds with α-pinene and β-pinene as the main components (Delazar et al., 2004; Barrero et al., 2005; Sharifi and Hazell, 2011; Benabderrahmane et al., 2016; Benabdallah et al., 2017; Ellahi et al., 2019). In addition, analysis of the monosaccharides has reported the majority presence of arabinose, galactose, glucose, rhamnose and xylose, while the most abundant aminoacids are aspartic acid, glutamic acid, histidine, proline and serine (Mirahmadi et al., 2019). Finally, triterpenoids like morolic acid, oleanonic acid, oleanolic acid, isomasticadienonic acid, 3-epi-isomasticadienolic acid, masticadienonic acid, dihydromasticadienonic acid, 3-*O*-acetyl-3epi (iso)masticadienolic acid, masticadienolic acid, dihydromasticadienolic acid, 3-acetoxy-3-epiisomasticadienolic acid, and 3-acetoxy-3-epimasticadienolic acid, ursonic acid) have also been identified (Sharifi, 2006).

In the TPM, various parts of *P. atlantica* have been utilized for the management of various skin disorders (like skin wounds, eczema, scabies, and lip fissures), gastrointestinal diseases (dyspepsia, peptic ulcer, esophagitis, and anal fissures), seizure, tremor, headache, asthma, pneumonia, hepatitis, and kidney diseases (Amin, 2005; Aghili, 2009; Bozorgi et al., 2013; Mahjoub et al., 2018). Recent reports have showed the extensive pharmacological and biological properties exhibited by different parts of *P. atlantica*, encompassing wound-healing, anti-inflammatory, antioxidant, nipple fissure healing, antimicrobial, antifungal, analgesic, anticancer, anticholinesterase, antidiabetic, hepatoprotective, antihypertensive, and antihyperlipidemic activities (Mahjoub et al., 2018; Pekacar and Deliorman Orhan, 2022).

Despite the critical role of novel preclinical studies in providing novel formulations and therapeutic agents in wound healing, there are some limitations. For instance, there are physiological and anatomical differences between animals and humans. Additionally, no study can recapitulate the complexity and heterogeneity of chronic wounds in humans. The lack of procedure standardization and translation of preclinical data into clinical models remains a crucial challenge (Grada et al., 2018).

Providing other preclinical models could confirm our findings in different models of burn and other scars. Preclinical reports could not only evaluate the wound healing potential of *PAK*, but also assess related scar vanishing effects. Future reports are needed to provide well-controlled clinical trials regarding evaluating the effectiveness of PVA/*PAK* in humans. In line, the development of *PAK*-based nanofiber dressings in humans could pave the road to wound healing. Further studies are also needed to provide other novel topical formulations to introduce products with a higher efficacy and a lower side effect.

## 5 Conclusion

In conclusion, our study has revealed the wound-healing potential of PVA/*PAK* nanofibers in two rat models of burn and excision wound repair. Moreover, we have reported the underlying mechanism through which these nanofibers show their therapeutic effects. Specifically, our results indicated antioxidative capacity of the nanofibers, as evidenced by the elevation in CAT and GSH levels in serum samples collected from the treated rats, plays a crucial role in facilitating the healing process. However, it is crucial to carry out further experimental and clinical investigations to confirm the efficacy of PVA/*PAK* nanofibers as a viable treatment option for wound healing.

## Data Availability

The datasets generated during and/or analyzed during the current study are available from the corresponding author upon reasonable request.

## Abbreviations

ANOVA, analysis of variance; CAT, catalase; CS, chitosan; DTNB, 5′-dithiobis-(2-nitrobenzoic acid); ECM, extracellular matrix; GPx, glutathione peroxidase; IFNs, interferons; ILs, interleukins; NO, nitric oxide; OD, optical density; PAK, *Pistacia atlantica* Desf. subsp. *kurdica* (Zohary) Rech. f.; PBS, phosphate buffered saline; PDGF, platelet-derived growth factor; fibroblast growth factor; PEO, poly-ethylene oxide; PMNs, polymorphonuclears; PVA, polyvinyl alcohol; SEM, Scanning electron microscopy; ROS, reactive oxygen species; SOD, superoxide dismutase; TGF-β, transforming growth factor-β; TNB, 5′-thio-2-nitrobenzoic acid; TNF-α tumor necrosis factor-α; TPM, Traditional Persian Medicine; ECM, extracellular matrix; VEGF, vascular endothelial growth factor.
